# Improved Less Invasive Surfactant Administration Success in Preterm Infants after Procedure Standardization

**DOI:** 10.3390/children8121145

**Published:** 2021-12-06

**Authors:** Björn Liebers, Chinedu Ulrich Ebenebe, Monika Wolf, Martin Ernst Blohm, Eik Vettorazzi, Dominique Singer, Philipp Deindl

**Affiliations:** 1 Department of Neonatology and Pediatric Intensive Care Medicine, University Children’s Hospital, University Medical Center Hamburg-Eppendorf, 20240 Hamburg, Germany; Bjoern.Lie@gmx.de (B.L.); c.ebenebe@uke.de (C.U.E.); m.wolf@uke.de (M.W.); m.blohm@uke.de (M.E.B.); d.singer@uke.de (D.S.); 2 Institute of Medical Biometry and Epidemiology, University Medical Center Hamburg-Eppendorf, 20240 Hamburg, Germany; e.vettorazzi@uke.de

**Keywords:** less invasive surfactant administration, respiratory distress syndrome, quality improvement, neonate

## Abstract

Less invasive surfactant administration (LISA) has been introduced at our tertiary Level IV perinatal center since 2016 with an unsatisfactory success rate, which we attributed to an inconsistent, non-standardized approach and ambiguous patient inclusion criteria. This study aimed to improve the LISA success rate to at least 75% within 12 months by implementing a highly standardized LISA approach combined with team training. The Plan Do Study Act method of quality improvement was used for this initiative. Baseline assessment included a review of patient medical records 12 months before the intervention regarding patient characteristics, method success rate, respiratory, and adverse outcomes. A multi-professional team developed a standardized LISA approach and a training program including an educational film, checklists, pocket cards, and team briefings. Twenty-one preterm infants received LISA before and 24 after the intervention. The mean LISA success rate improved from 62% before the intervention to 92% (*p* = 0.029) after the intervention. Implementing a highly standardized LISA approach and multi-professional team training significantly improved the methods’ success rate.

## 1. Introduction

Respiratory distress syndrome (RDS) is a major cause of morbidity in preterm infants. Non-invasive respiratory support and early surfactant replacement are cornerstones of RDS therapy [1]. In the last decade, methods for the endotracheal administering of surfactant using a thin catheter under laryngoscopy, with the infant spontaneously breathing on CPAP, are emerging and have been described and called less invasive surfactant administration (LISA) or minimally-invasive surfactant administration (MIST) [2]. LISA requires a fundamental change in the delivery room workflow while stabilizing a preterm infant with respiratory distress syndrome.

Several randomized trials and meta-analyses [3,4,5,6] comparing LISA to surfactant therapy via an endotracheal tube and with the intubation surfactant and extubation (InSurE) procedure suggest that LISA is superior in reducing the need for mechanical ventilation and the combined outcome of death or bronchopulmonary dyplasia [7]. In addition, Bugter et al. found that patients who had received LISA were exposed to fewer interventions, such as radiographs and blood gas analyses [8]. LISA might even reduce the total number of preterm infants needing any mechanical ventilation during hospitalization [4]. The evidence for these observations is, however, not yet clear. The centers that participated in the trials performed the LISA procedure according to different local protocols [9], one of the studies’ concerns. Several studies have shown that the introduction of LISA had positive effects compared with historical comparison groups treated conventionally, including reductions in the need for oxygen, mechanical ventilation, and surfactant [10,11,12].

International and national surveys indicate a lack of training and guidelines [13], heterogenous approaches to surfactant replacement therapy, no clear indications for LISA, and that overall respiratory support strategies for preterm infants, in general, vary widely [14,15,16,17,18]. Critical voices also question LISA as a therapeutic concept and consider the data insufficient to recommend this procedure universally [19]. Another unresolved problem is sedation during laryngoscopy using the LISA procedure [20,21]. LISA failure rates can be as high as about one in four patients [22]. The lack of standardization and guidelines lead to the problem that the term LISA presumably covers a wide range of different approaches concerning different surfactant catheters, surfactant amount and preparation, respiratory monitoring, patient positioning, premedication regimens, and additional supportive measures [23,24].

### 1.1. Problems with LISA Prior to the Intervention

We introduced LISA in our tertiary University Level IV Perinatal Center in 2016 to stabilize preterm infants with RDS in the delivery room. At that time, no approved LISA catheters were available in Germany. We applied LISA sporadically following different approaches depending on the neonatologist in charge. Several different gastric tubes were used as LISA catheters, binasal, and mononasal continuous positive airway pressure was applied with inconsistent settings. There was no standardization regarding the patient inclusion criteria, team briefing, equipment, and abortion criteria. The team was uncertain about performing LISA because of vague instructions and concerned about an unsatisfactory method’s success.

### 1.2. Aims

This study aimed to improve the LISA’s success to at least 75% within 12 months by implementing a quality improvement initiative based on rigorous LISA procedure standardization and a comprehensive multi-professional training program.

## 2. Materials and Methods

### 2.1. Interventions

The Plan Do Study Act (PDSA) method of quality improvement was used for this initiative, and the Revised Standards for Quality Improvement Reporting Excellence (SQUIRE 2.0) were applied to report the results [25]. A multi-professional team was formed, summarized the existing problems, interviewed staff, assembled, and tested different materials and approaches.

### 2.2. PDSA Cycles

In joint discussions and training on high-fidelity airway simulators (Premature AirwayPaul, SIMCharacters, Vienna, Austria, and Premature Anne, Laerdal Medical, Stavanger, Norway), the multi-professional team of physicians and nurses developed a highly standardized LISA concept. During this preliminary training on the simulator, the exact distribution of tasks in the delivery room, the spatial arrangement of persons and materials, the type of monitoring, and the positioning of the patient (Figure S1) were specified. The tasks in preparing the material were divided between both professional groups. In addition, a LISA catheter was selected from several alternative products (LISAcath, Chiesi, Hamburg, Germany, Chiesi, Hamburg, Germany) and the Surfactant administered (Surfactant: Curosurf, Chiesi, Hamburg, Germany) was defined. In addition, the team agreed on patient characteristics for children eligible for the LISA procedure (birth weight ≥ 700 g, gestational age ≥ 25 + 0 weeks) and on abortion criteria in the interest of patient safety (Figure S1). After both parents informed consent, we filmed a preterm infant’s stabilization in the delivery room according to the standardized LISA procedure and produced an educational film demonstrating the procedure step by step (Figure S1). In addition, members of the medical team created laminated pocket cards, mnemonic aids, posters for the delivery room (Figure S1). The staff was trained from February 2020 to April 2020 using the educational film and slide presentations with question and answer sessions in each professional group. During the educational phase, we received team feedback and expanded the checklists and the educational film to include: 1. The exact timing of each LISA step, 2. Following diagnostic steps in the neonatal intensive care unit (NICU), 3. Timing of the placement of a feeding tube. After more procedures were completed, we introduced a structured team briefing to improve preparation for the LISA procedure and checklists for the delivery room equipment.

### 2.3. Measures

We extracted data from our electronic medical record system from all preterm infants who received surfactant via LISA procedure in the delivery room before the intervention between February 2018 and February 2020 and after the intervention from May 2020 to February 2021.

### 2.4. Process Measure

The process measure of our intervention was the LISA success rate. We defined LISA failure as intubation within the following 72 h, the need for a second surfactant dose, or abortion of LISA procedure due to complications. The indication for intubation was FiO_2_ requirement > 0.4% or FiO_2_ > 0.3% in combination with dyspnea. If a second surfactant dose was needed, it was administered via intubation, as we initially introduced LISA as part of delivery room stabilization.

### 2.5. Balancing Measures

We compared patient characteristics and prenatal information and analyzed in-hospital outcomes, including time on invasive and non-invasive ventilation, the duration of parenteral nutrition, and the number of red blood cell transfusions before and after the intervention to account for potential changes. We also compared therapy duration with inotropic medication, the incidence of oxygen requirement with a corrected gestational age of 36 weeks, retinopathy, necrotizing enterocolitis, intraventricular hemorrhage > grade II, and death between the two time periods. Figure S2 displays the aim, the key drivers, the interventions, and the process and balancing measures of this quality improvement initiative.

### 2.6. Statistical Analysis

Continuous variables were reported as mean ± standard deviation (SD), categorical variables as category counts and percentages. We compared count data using the Fishers’ exact tests and the Wilcox-test to compare continuous variables between both periods. *p*-values less than 0.05 were considered significant. We calculated a multivariate logistic regression model to analyze the effects of gestational age, birth weight, 5-min APGAR, mode of delivery, and lung maturation induction on LISA procedure failure. Statistical analyses were performed using R 4.1.1 (R Core Team, Vienna, Austria).

## 3. Results

We received a waiver for this study from the local ethics committee (WF-157/20). The data of 21 preterm infants that received surfactant via LISA before and 24 after the intervention were retrospectively analyzed. Patients were similar regarding prenatal characteristics, mode of delivery, postnatal stability, and drug dosages during stabilization in the delivery room (Table 1). However, due to the specification of our local inclusion criteria for the LISA procedure as part of the standardization process, the patients after the intervention had a significantly higher mean gestational age, birth weight, and body length. Table 1 shows details regarding the patient characteristics.

**Figure 1:**
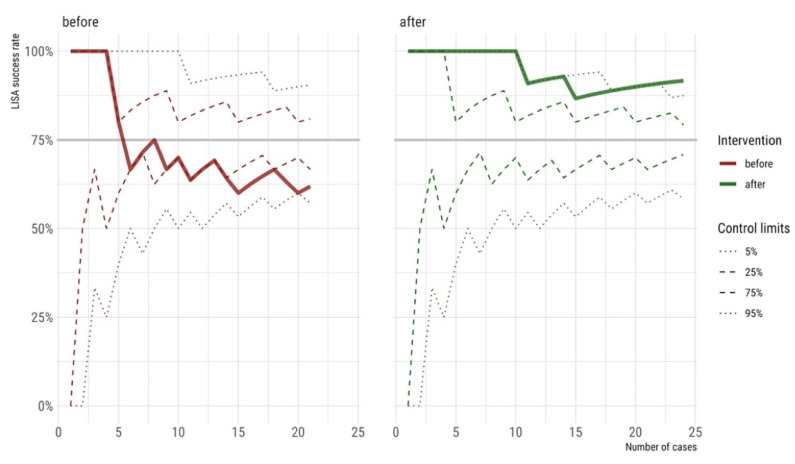
Process control chart: The less-invasive surfactant administration (LISA) success rates were calculated cumulatively with the respective 95% control limits using the Clopper-Pearson method. The light grey horizontal line indicates the target LISA success rate of 75%.

We generated a process control chart to plot the LISA success rate over time before and after the intervention together with the target success rate of 75% (Figure 1). Before the intervention, the mean LISA success rate was 62%. After the intervention, the mean success rate significantly raised to 92% (*p* = 0.029) (Table 1 and Figure 1). In addition, after the intervention, significantly less mean ± SD time passed between two consecutive LISA procedures than before (11.8 ± 15.5 days vs. 36.1 ± 37.4, *p* < 0.001).

### 3.1. LISA Failure Causes

**Figure 2:**
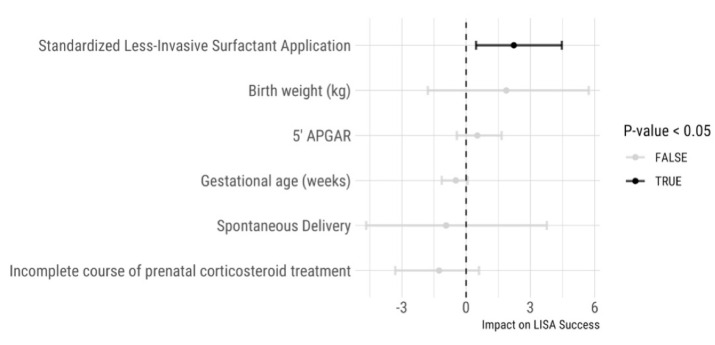
Logistic multivariable regression model to predict less invasive surfactant application success. Only the time point after the intervention was a significant predictor of successful less invasive surfactant application.

We analyzed the reasons for LISA failure. Table 1 shows the percentage of patients before and after the intervention requiring additional surfactant administration, intubation < 72 h after LISA, and the cases in which the LISA procedure was aborted. After the intervention, the respective failure causes did not occur significantly less frequently. The inclusion criteria defined during the LISA procedure standardization process resulted in a significant difference regarding the mean gestational age, size, and length of patients in the two periods studied. We, therefore, calculated a logistic regression model to analyze the influence of gestational age, birth weight, 5-min APGAR, completeness of prenatal corticosteroid course, mode of delivery, and the study period on the LISA success rate. The logistic regression identified the intervention as the sole significant predictor of a higher LISA success rate (Figure 2, *p* = 0.024). None of the other potential factors had a relevant influence on the LISA success rate in our patient population.

### 3.2. Balancing Measures—In-Hospital Outcomes

The comparison of the in-hospital outcome parameters before and after the intervention showed a similar mean ± SD time on invasive ventilation, non-invasive ventilation, and length of stay. In addition, the number of transfusions 0.8 ± 1.2 vs. 0.1 ± 0.3, (*p* = 0.054), and duration of inotropic support (0.8 ± 2.8 vs. 0.1 ± 0.4 days, *p* = 0.128) were similar. However, the duration of parenteral nutrition was significantly shorter after the intervention: 17.8 ± 7.3 vs. 14.8 ± 3.6 days (*p* < 0.001). The adverse long-term outcome parameters were similar in both periods: high-grade intraventricular hemorrhage, retinopathy of prematurity, necrotizing enterocolitis requiring surgical intervention, oxygen requirement at 36 weeks gestational age, and death (Table 1).

## 4. Discussion

After an intervention, we report a significant improvement in the LISA success rate, including rigorous procedure standardization and multi-professional team education. Despite strict failure criteria, we observed a consistently very high success rate of 92% successful LISA applications compared to other studies.

### 4.1. LISA Success

The changes in the local inclusion criteria for preterm infants qualifying for LISA (birth weight ≥ 700 g, gestational age ≥ 25 weeks) as part of the intervention resulted in more mature and heavier preterm infants on average after the intervention compared to before (Table 1). A logistic regression model showed that neither gestational age nor birth weight significantly influenced LISA success. Thus, only the timing after the intervention was critical for LISA’s success (Figure 2). We conclude that the intervention led to a significant increase in the LISA success rate in our patient population.

LISA was only considered successful if no intubation was necessary within 72 h and no second surfactant dose was administered. We chose this success definition because the second surfactant dose was always administered via endotracheal intubation in our institution. Despite this stringent definition, we achieved a high mean LISA success rate of 92% after the intervention compared with other studies that reported success rates of around 75% [4] or 80% [26]. We introduced LISA as a primary surfactant replacement therapy method in preterm infants with RDS during delivery room care. LISA in the delivery room care setting could theoretically have a higher success rate than LISA in the NICU because laryngoscopy performed without medication may be easier in infants who are just born compared to those that are already a few hours old presenting with a higher muscle tone.

After the intervention, no LISA procedure had to be aborted. The training of the entire team may have led to more confidence in the handling of the necessary devices and an overall gentler initial care of the infants receiving LISA. Recent studies showed that guidelines and training of operators are helpful to implement LISA safely and successfully in daily routine [26,27].

### 4.2. Procedure Frequency

After the intervention, significantly less time between two LISA applications elapsed, and the success rate settled at a high level of 92% (Figure 1). Complex clinical procedures succeed more often when applied with high frequency [28]. In addition, perinatal centers with very high patient volumes often also achieve better outcomes [29]. Due to the higher LISA frequency after the intervention, the team was more practiced and confident and thus likely more successful.

### 4.3. Procedure Standardization

Standardization of LISA with transparent procedures, task distribution, responsibilities, stability criteria, discontinuation criteria, and a defined target group and equipment specifications, as we had established for this complex procedure, might have improved team confidence and overall performance. Given the small number of cases, the transferability of our results is limited, but there are evident effects that should be confirmed in more extensive studies. Roberts et al. reported a higher LISA success rate when clinicians with greater competence in endotracheal intubation performed the procedure [30]. In our study, the LISA procedure was performed only by senior physicians with extensive experience in endotracheal intubation of preterm infants.

### 4.4. Teaching Medical Procedures in Large Multi-Professional Teams

We used an educational film to train a large multi-professional NICU medical team in a uniform and resource-efficient manner within a relatively short time. Demonstration of clinical skills is superior to the simple description because they engage multiple cognitive and psychomotor skills [31]. In addition, experienced health care professionals often perform clinical skills subconsciously, which is why they often have difficulty breaking down the process into steps when teaching skills [32]. Video teaching, which allows step-by-step demonstration, is one effective way to teach complex medical skills [31]. For example, a study by Hawkes et al. reported that video teaching showing the neonatal intubation procedure and additional information improved the understanding of intubation and the time needed to perform neonatal intubation correctly [33].

### 4.5. Balancing Measures—In-Hospital Outcomes

We examined several in-hospital outcomes to analyze whether the intervention changed the outcome of infants who received LISA. Except for a shorter duration of parenteral nutrition after the intervention, the duration of invasive and noninvasive ventilation, the number of blood transfusions, and the duration with inotropic support were similar. Adverse events also occurred with similar frequency after the intervention compared to before (Table 1). We attribute the shorter duration with parenteral nutrition to the higher gestational age of the patients after the intervention. We conclude that the intervention did not significantly change the outcome of the infants.

### 4.6. Limitations

Our study has several limitations. We analyzed the outcome of patients in only one level IV university perinatal center. Thus, comparison between two time periods may potentially introduce bias. The numbers of LISA patients are relatively small, so the observations should be interpreted with caution. By defining the patient population as having a gestational age greater than 25 weeks and a minimum birth weight of 700 g, the patients in the post-intervention group were larger and had a higher gestational age. In addition, fewer patients with intrauterine growth restriction or small for gestational age received LISA. Thus, the difference between the groups could have confounded the improved LISA success rate. However, we accounted for these group differences by calculating a logistic multivariate regression model including both birth weight and gestational age as predictors. Nevertheless, we cannot exclude that infants who received LISA after the intervention were healthier than before. Finally, as we exclusively applied LISA directly after birth, our results are not directly transferable to LISA application in the NICU.

## 5. Conclusions

A quality improvement initiative consisting of the implementation of a highly standardized LISA approach and extensive multi-professional team training significantly improved the methods’ success rate in our level IV perinatal center.

## Figures and Tables

**Table 1:** Patient characteristics and in-hospital outcome before and after the intervention.

Variable	Before	After	*p*-Value	CI
	N = 21	N = 24		
**Patient characteristics**				
Birth weight (kg)	1073.3 ± 456.9	1277.7 ± 327.3	**0.022**	(−470–−40)
Gestational age (weeks)	28.4 ± 2.6	29.5 ± 1.8	0.115	(−3–0)
Height (cm)	36.1 ± 4.3	38.9 ± 3.2	**0.005**	(−5.5–−1)
Head circumference (cm)	26.0 ± 3	27.6 ± 2	**0.034**	(−3–0)
5′-APGAR	7.8 ± 1	8.1 ± 0.8	0.261	(−1–0)
Caffeine citrate dose in DR (mg/kg)	20.2 ± 2	19.0 ± 1.6	0.087	(0–2)
Surfactant dose (mg/kg)	193 ± 62	189 ± 48	0.882	(−33.7–32.9)
Delivery Mode				
Cesarean Section	20 (95)	22 (92)	1	(0.09–112)
Spontaneous Delivery	1 (5)	2 (8)	1	(0.08–17.4)
Multiple gestation	11 (52)	16 (67)	0.374	(0.14–2.15)
Amniotic infection	3 (14)	2 (8)	0.652	(0.19–23.9)
IUGR	5 (24)	1 (4)	0.083	(0.68–353)
SGA	6 (29)	2 (8)	0.121	(0.65–48.7)
Incomplete course of prenatal corticosteroid treatment	7 (33)	9 (38)	1	(0.2–3.4)
**Less invasive surfactant administration**				
Success	13 (62)	22 (92)	**0.029**	(0.01–0.94)
Delta FiO_2_ after LISA	0.2 ± 0.1	0.2 ± 0.2	0.18	(−0.16–0.03)
Medication during LISA	3 (14)	0 (0)	0.093	(0.49–Inf)
Additional surfactant administration	5 (24)	1 (4)	0.083	(0.68–353)
Intubation < 72 h after LISA	7 (33)	2 (8)	0.061	(0.85–59.4)
LISA aborted	2 (10)	0 (0)	0.212	(0.21–Inf)
**Respiratory outcomes**				
Invasive ventilation in the first week	7 (33)	2 (8)	0.061	(0.84–59.4)
Invasive ventilation (days)	2.4 ± 6	0 ± 0.1	0.09	(0–0.14)
Non−invasive ventilation (days)	26.7 ± 18.4	18.2 ± 12.4	0.125	(−2.37–18.7)
Additional oxygen requirement at 36 weeks of gestation	1 (5)	0 (0)	0.467	(0–Inf)
Air leak	3 (14)	1 (4)	0.326	(0–209)
**Adverse outcomes**				
Death	1 (5)	0 (0)	0.467	(0.03–Inf)
IVH > Grade II	0 (0)	2 (8)	0.491	(0–6.1)
NEC Surgery	1 (5)	0 (0)	0.467	(0.03–Inf)
ROP	1 (5)	0 (0)	0.467	(0.03–Inf)
Length of stay	56 ± 28.1	51.8 ± 13.2	0.937	(−11.42–16.4)

Categorical variables are shown as counts (percentage), *p*-values were calculated using a two-sided Fisher’s Exact Test for Count Data, continuous variables are shown as mean ± standard deviation, *p*-values were calculated using a two-sided Wilcox-Test. CI: confidence interval, DR: delivery room, Inf: Infinite, IUGR: Intrauterine growth restriction, IVH: intraventricular hemorrhage, SGA: small for gestational age, NEC: necrotizing enterocolitis, ROP: retinopathy of prematurity.

## Data Availability

The data presented in this study are available on request from the corresponding author.

## Supplementary Materials

The following are available online at https://www.mdpi.com/article/10.3390/children8121145/s1, Figure S1: Less-invasive surfactant administration procedure standard, Figure S2: Key Driver Diagram with the Specific, Measurable, Applicable, Realistic, and Timely (SMART) Aim, the interventions, process measure and balancing measures as part of this quality improvement initiative.
