# Supplementary material for: Improved Less Invasive Surfactant Administration Success in Preterm Infants after Procedure Standardization
Source: Children (Basel). 2021 Dec 6;8(12):1145. doi: 10.3390/children8121145 (PMC8700472; doi:10.3390/children8121145)
Supplement: Supplementary file 1 [file children-08-01145-s001.zip › Figure_S1_LISA_revision.pdf]

Department of Neonatology and Pediatric Intensive Care Medicine
University Children's Hospital
University Medical Center Hamburg-Eppendorf

Universitätsklinikum Hamburg-Eppendorf

Procedure Standard

Less Invasive Surfactant Administration – LISA

1. Aims

- Surfactant administration during spontaneous breathing
- Stress reduction
- Optimized thermal management

2. Inclusion Criteria

- >= 25+0 – 33+6 weeks of gestation
- >= 700 g birth weight
- Spontaneous breathing after 10–15 minutes under CPAP
- FiO_2 > 0.4, or FiO_2 > 0.3 *and* dyspnea
- SpO_2 >= 90 %, max. FiO_2 0.7

3. Preparation (see checklists)

- Thermal management (doors closed)
- Resuscitation unit (Fig. 1A)
- Plastic sheet
- Hygiene measures: always: mouth guard, sterile gloves for intubation; for ≤28 weeks gestation: additional gown).
- Caffeine citrate 20 mg/kg body weight as a short infusion over 5 min.
- CPAP 8 L/min. Flow, PEEP 10 cmH_2O
- Set FiO_2 0.21-0.3
- Preparation of LISA table (Fig. 1B)
- Mononasal CPAP: shorten tube to 10 cm (sterile) (Fig. 1C)

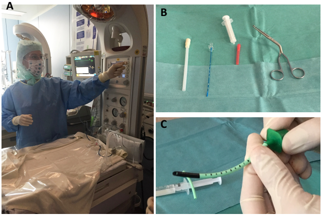

Figure 1: A: Preparation of resuscitation unit (nursing team), B: Preparation of sterile LISA material (physician team), C: Shorten tube to 10 cm (physician team).

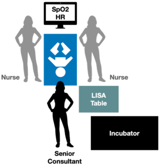

Figure 2: Distribution of tasks

4. Team Briefing

- Medical history
- Patient information
- Expected risks
- Precise allocation of tasks (Fig. 2)
- Plan alternative(s) to LISA procedure

Optimal timing of LISA

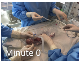

Mask-CPAP

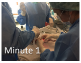

SpO_2 Monitoring, right arm

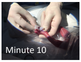

i.v. access, Blood collection / Blood gas

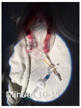

Caffeine citrate, as a short infusion over 5 min.

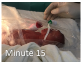

Mononasal CPAP

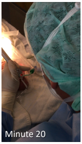

Laryngoscopy

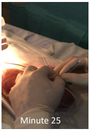

LISA Catheter Placement
1-2 cm insertion depth

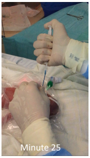

Surfactant administration, in small aliquots over 1-3 minutes

What happens next?

- Gastric tube (~minute 30)
- Binasal CPAP (~minute 35)
- Transfer to the incubator (~minute 40)
- Contact with parents (~minute 50)
- Transport to the NICU (~minute 60)

NICU

- Chest radiography
- Early tCO_2/tO_2 monitoring
- blood gas analysis

Abortion criteria

- Apnea > 30 sec.
- Desaturation < 50% for > 30 sec.
- Bradycardia < 80/min. for > 30 sec.
- FiO_2 > 0.7
- No safe laryngoscopy possible
- Difficulty with LISA catheter placement
- Clinical discretion of the senior consultant

Consent for publication from patient's parents and staff have been obtained.
