# Supplementary material for: Improved Less Invasive Surfactant Administration Success in Preterm Infants after Procedure Standardization
Source: Children (Basel). 2021 Dec 6;8(12):1145. doi: 10.3390/children8121145 (PMC8700472; doi:10.3390/children8121145)

Department of Neonatology and Pediatric Intensive Care Medicine
University Children's Hospital
University Medical Center Hamburg-Eppendorf
Germany

Universitätsklinikum Hamburg-Eppendorf

Bjoern Liebers, Chinedu Ulrich Ebenebe, Monika Wolf, Martin Ernst Blohm, Eik Vettorazzi, Dominique Singer, and Philipp Deindl

Less-invasive surfactant administration (LISA): A quality improvement initiative

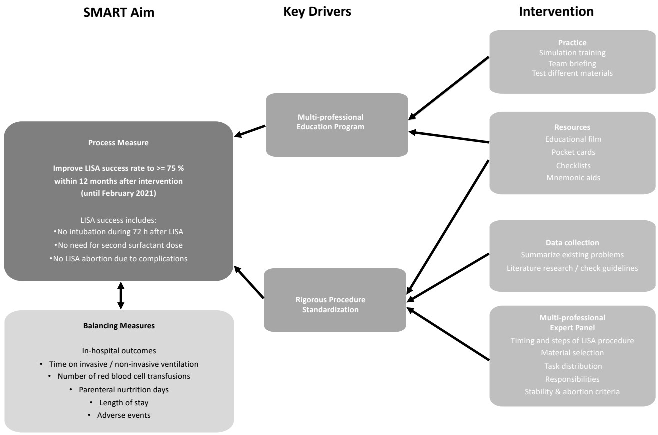

Supplement: Supplementary file 1 [file children-08-01145-s001.zip › Figure_S2_Key_Drivers_Diagram.pdf]
